# Development of a Rapid and Simple Method for Detection of Protein Contaminants in Carmine

**DOI:** 10.1155/2015/748056

**Published:** 2015-03-29

**Authors:** Norihisa Nakayama, Yutaka Ohtsu, Daisuke Maezawa-Kase, Ken-Ichi Sano

**Affiliations:** ^1^Graduate School of Environmental Symbiotic System and Department of Innovative Systems Engineering, Nippon Institute of Technology, 4-1 Gakuendai, Miyashiro-machi, Saitama 345-8501, Japan; ^2^Kishi Kasei Company, 1-10-8 Fukuura, Kanazawa-ku, Yokohama 234-0004, Japan

## Abstract

Protein contaminants in carmine can cause dyspnea and anaphylactic reactions in users and consumers of products containing this pigment. The method generally used for detection of proteins in carmine has low reproducibility and is time-consuming. In this study, a rapid, simple, and highly reproducible method was developed for the detection of protein contaminants in carmine. This method incorporates acidic protein denaturation conditions and ultrafiltration. To prevent protein aggregation, sodium dodecyl sulfate containing gel electrophoresis running buffer was used for dispersing the carmine before filtration. An ultrafiltration device was used to separate the protein contaminants from carminic acid in the carmine solution. Two ultrafiltration devices were compared, and a cylindrical device containing a modified polyethersulfone membrane gave the best results. The method had high reproducibility.

## 1. Introduction

**Figure 1:**
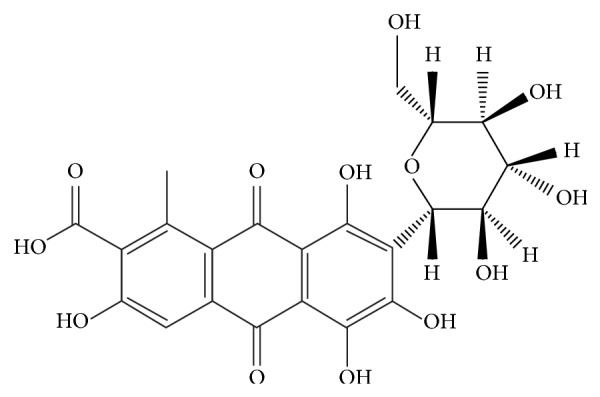
Carminic acid.

Carmine is a natural red pigment extracted from dried scale insects (cochineal,* Dactylopius coccus*) [1]. The main chemical component of this pigment is carminic acid (7-*α*-d-glucopyranosyl-9,10-dihydro-3,5,6,8-tetrahydroxy-1-methyl-9,10-dioxoanthracene carboxylic acid, Figure 1). Carmine is widely used for coloring food products, cosmetics, and medicines [2, 3]. In 2012, the Japanese Consumer Affairs Agency published a report on carmine detailing its potential links to dyspnea and anaphylactic reactions [4]. This report detailed approximately 20 articles from 1960s about anaphylaxis resulting from the use of the cosmetics or consumption of food containing carmine. Carminic acid is not the cause of these allergic reactions; they are rather caused by protein contaminants in the carmine [5, 6]. Some of the allergenic protein contaminants have been identified, and one of them has high homology with phospholipase A, which is a well-known allergen in vespidae venom [7]. Hence, the use of carmine in foods is regulated both in Japan and European Union.

For consumer safety, allergenic protein contaminants in carmine need to be monitored. The general method for detection of protein contaminants in carmine involves suspension of the pigment in an acidic solution (1% aqueous solution of phosphoric acid) and then separation of any protein contaminants from the solution by HPLC [8]. The collected proteins are identified by SDS-PAGE [9]. This method is time-consuming because about ten HPLC analyses are required to obtain a sufficient quantity of protein for SDS-PAGE analysis. Furthermore, the reproducibility of the HPLC separation is low. Therefore, an alternative method that is rapid, simple, and has high reproducibility is needed. In this study, we developed a rapid and simple method for detection of protein contaminants in carmine with high reproducibility.

## 2. Materials and Methods

Three samples of dry carmine were used. Sample A was purchased from Biocon (cochineal extract, lot number M008363). Sample B was manufactured by Kishi Kasei Company (carminic acid, lot number 174302), and we carried out further purification of Sample B as follows. Firstly, Sample B was dissolved in 5% of phosphoric acid and loaded on a spherical polymerized divinylbenzene resin. Then bound carminic acid was eluted by 50% ethanol and dried (Sample C).

To separate the protein fraction from the carminic acid, we used an ultrafiltration device with a molecular weight cut-off of 3,000 Da. Two ultrafiltration devices were tested; one was v-shaped, with a regenerated cellulose membrane (Amicon Ultra-0.5 mL 3 K, Millipore, Billerica, MA), and the other was cylindrical, with a modified polyethersulfone membrane (Nanosep 3 K Omega, Pall Corporation, Port Washington, NY). The initial sample volume was 500 *μ*L, and the filtration was done by centrifugation until the samples were concentrated to about 0.4–0.7 of their initial volume. The time for centrifugation depended on each samples. The protocol was repeated more than 14 times. After these filtration steps, the concentration of carminic acid in each sample would be diluted by at least 6 × 10^4^ times mathematically.

Electrophoresis was carried out using precast gels (15% Choju Gel, Oriental Instruments, Sagamihara, Japan) and stained using Silver Stain MS kit (Wako Chemicals, Osaka, Japan) [10].

## 3. Results and Discussion

### 3.1. Removal of Carmine Acid from Cochineal Dispersed in Protein Denaturant Solution

**Figure 2:**
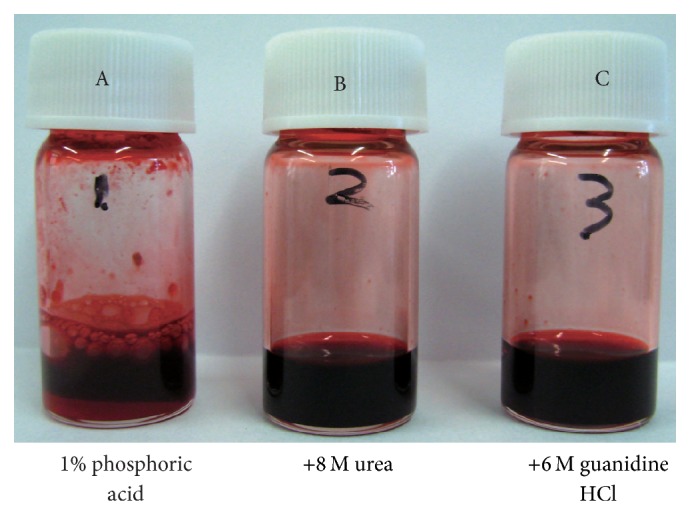
Carmine (Sample A, cochineal extract, Biocon) suspensions. (A) is suspended in 1% phosphoric acid. (B) is dispersed in 1% phosphoric acid and 8 mol/L urea. (C) is dispersed in 1% phosphoric acid and 6 mol/L guanidine hydrochloride.

Initially, we investigated the separation of proteins from the carmine solution. Three types of carmine were used, as detailed in Section 2. Each carmine sample was dispersed in phosphoric acid (1% (mass fraction) aqueous solution) at a final concentration of 50 mg/mL using ultrasonication. A white precipitate of aggregated protein contaminants was observed when Sample A (cochineal extract, lot number M008363, Biocon) was dispersed in phosphoric acid (Figure 2). We had confirmed low reproducibility of HPLC separations, such as disappearance and shift of a peak from contaminant proteins (data not shown). The aggregation of protein contaminants in the phosphoric acid would reduce the reproducibility of protein separation. As a logical approach to prevention of aggregation of protein contaminants, we made use of protein denaturant first. Urea and guanidine hydrochloric acid are commonly used for dissolution of protein aggregates. Therefore, in the present method, urea (8 mol/L) and guanidine hydrochloric acid (6 mol/L) were added to dissolve the precipitate (Figure 2).

Detection of proteins is usually performed by colorimetric methods such as biuret method and ninhydrin reaction. Free carminic acid interferes with the detection of allergenic proteins in the carmine by colorimetric analysis, and it needs to be removed before analysis. In addition, to detecting the allergenic proteins, protein fractions of >6 kDa need to be collected, and determining the molecular weight of protein contaminants is also important. Both SDS-PAGE and mass spectrometric analysis are generally used methods for determination of molecular weight of proteins. Bound carminic acid on protein shift in molecular weight is found in mass spectrometric analysis, and a smear band pattern is also acquired from SDS-PAGE. Consequently, in the present study, two ultrafiltration devices were compared for separating the protein fraction from the carmine solution. Both of these had molecular weight cut-offs of 3,000 Da and are described in Section 2.

**Figure 3:**
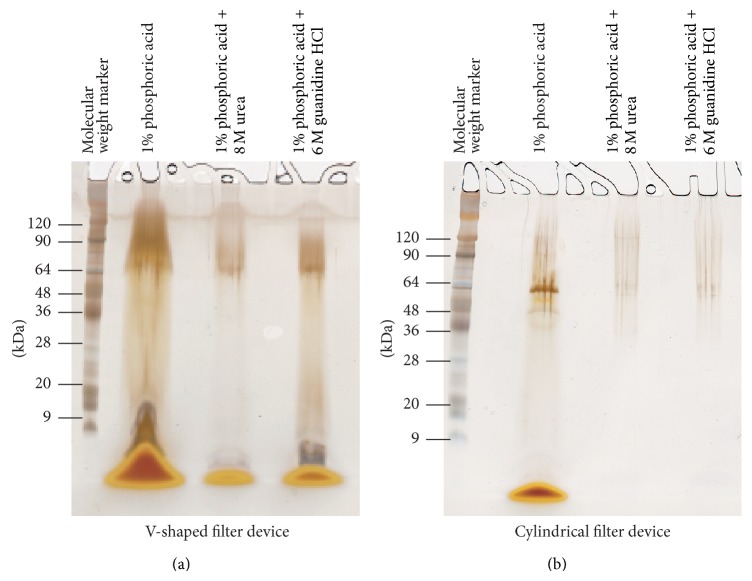
SDS-PAGE of protein contaminants in carmine (Sample A, cochineal extract, Biocon) after silver staining. (a) Amicon Ultra-0.5 ultrafiltration device. (b) Nanosep 3 K Omega ultrafiltration device.

The initial sample volume was 500 *μ*L, and the filtration was done by centrifugation until the samples were concentrated to about 0.4–0.7 of their initial volume. The protocol was repeated more than 14 times. The optimum initial concentration of carmine was investigated. 50, 100, and 200 mg/mL concentrations of carmine were suspended in each solution. At 100 and 200 mg/mL of carmine, carminic acid suspensions were difficult to remove by both ultrafiltration devices, and a concentration of 50 mg/mL was used in subsequent experiments. After filtration, the concentration of carminic acid in each sample was diluted by at least 6 × 10^4^ times. The Amicon Ultra-0.5 v-shaped ultrafiltration device could not quantitatively remove the carminic acid, and thus the SDS-PAGE results were obscured by remaining pigment (Figure 3(a)). However, the cylindrical ultrafiltration device did remove sufficient carminic acid to allow the protein contaminant band to be clearly observed at around 60 kDa. Although the cylindrical ultrafiltration device provided more effective removal of carminic acid than the v-shaped device, the detection of low molecular weight proteins (<20 kDa) was low (Figure 3(b)). These results indicate that the Nanosep 3 K Omega ultrafiltration device is better than the Amicon Ultra-0.5 for the removal of carminic acid, but protein recovery may be an issue.

### 3.2. Removal of Carmine Acid from Cochineal Dispersed in SDS-PAGE Running Buffer

The loss of the protein could be caused by damage to the ultrafiltration device from the high specific gravity solution used for dispersion of the carmine (e.g., 8 mol/L urea and 6 mol/L guanidine hydrochloric acid). Therefore, SDS-PAGE running buffer, 25 mmol/L tris(hydroxyamino)methane, 200 mmol/L glycine, and 1% (mass fraction) SDS, was trialed for dispersion of the carmine samples. This buffer has the following benefits to our analysis: it contains SDS, which is a surfactant having strong protein-denaturing ability; the specific gravity is much lower than that of 8 mol/L urea and 6 mol/L guanidine hydrochloric acid; and it does not interfere with protein migration in acrylamide gel.

**Figure 4:**
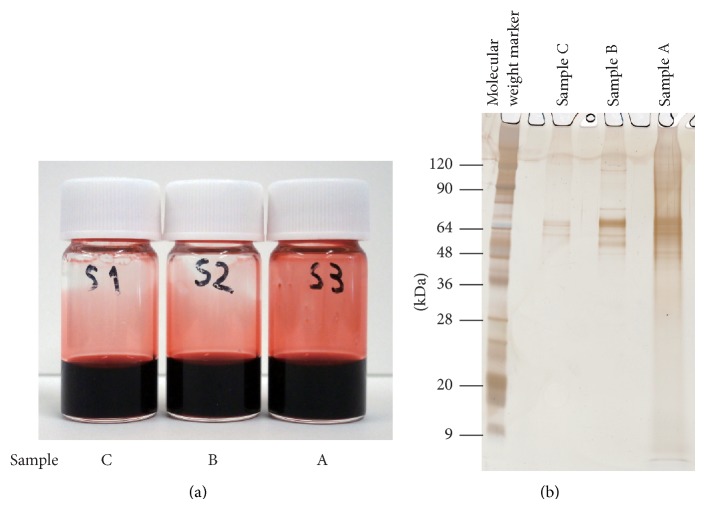
(a) Images of carmine suspensions in SDS-PAGE running buffer. Left: Sample C (carminic acid purified using divinylbenzene resin, Kishi Kasei). Middle: Sample B (carminic acid, Kishi Kasei). Right: Sample A (cochineal extract, Biocon). (b) SDS-PAGE results for protein contaminants in carmine with the Nanosep 3 K Omega.

The three carmine samples were each dispersed at a final concentration of 50 mg/mL in SDS-PAGE running buffer by ultrasonication (Figure 4(a)). The Nanosep 3 K Omega was used to remove carminic acid, and this was followed by SDS-PAGE and silver staining (Figure 4(b)). For Sample A, not all of the carminic acid was removed by ultrafiltration, but the remaining carminic acid had little effect on the electrophoresis. Several protein bands were observed at 50–70 kDa for all of the carmine samples. The intensities of these bands were highest in Sample A, followed by Sample B, and lowest in Sample C. Protein bands were also observed in a lower molecular weight region (<20 kDa) for the partially purified carmine. We confirmed the results of the rank order of intensities of protein bands' exhibited high reproducibility.

**Figure 5:**
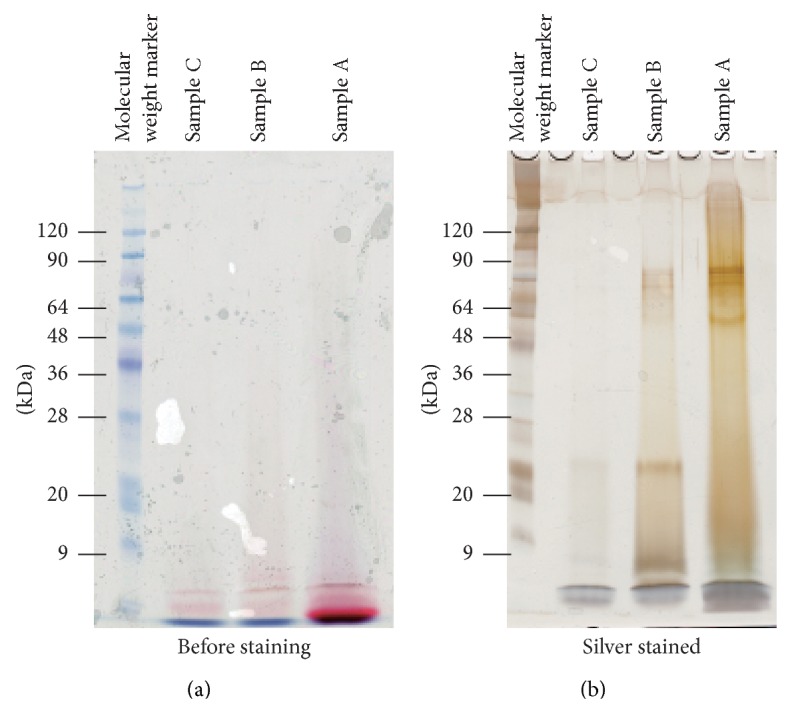
(a) Image of acrylamide gel immediately after SDS-PAGE for protein contaminants in carmine with the Amicon Ultra-0.5. (b) Silver stained.

The Amicon Ultra-0.5 was also tested for removing carminic acid from a suspension of carmine in SDS-PAGE running buffer. Compared with the Nanosep 3 K Omega device, much of time was required (several days) and more of the carminic acid remained in the solution after Amicon Ultra-0.5 filtration. Remaining carminic acid was able to be confirmed by view of acrylamide gel before staining (Figure 5(a)) and the electrophoretic pattern of protein contaminants showed more smearing by binding carminic acid on proteins (Figure 5(b)). Based on the SDS-PAGE results, the dependence on purity of carmine was obvious with the Nanosep 3 K Omega protocol. Although the detection limit of the low molecular weight proteins (<20 kDa) seemed to be lower with the Nanosep 3 K Omega than with the Amicon Ultra-0.5, all of the bands were clearly visible still. Also the proteins detected at low molecular weight (<20 kDa) by silver staining with the Amicon Ultra-0.5 were also possible artifact due to the effect of the remaining carminic acid. To examine the effect of the shape of the ultrafiltration device on the isolation and detection of the protein contaminants, we also tested a Microsep Advance Centrifugal Device 3 K (Pall Corporation). Like the Nanosep 3 K Omega, the Microsep device contains a modified polyethersulfone membrane. However, this device is v-shaped rather than cylindrical, and the membrane is 10 times larger than that in the Nanosep; filtration with this device took a long time (several days). The loss of low molecular weight proteins was similar to that with the Nanosep 3 K Omega (data not shown). These results suggest that the cylindrical filtration device is more suitable for removal of carminic acid than the v-shaped ultrafiltration device.

Carminic acid can form strong bonds with proteins through both electrostatic and hydrophobic interactions. Therefore, separation of carminic acid from protein contaminants is technically difficult. The method commonly used for detection of protein contaminants in carmine involves suspension of the carmine in an acidic solution. The acidic solution results in protein denaturation and protein aggregation, which cause analytical and separation difficulties. In this study, we found that SDS-PAGE running buffer worked as an effective solvent for dispersion of carmine for removal of protein contaminants. The ultrafiltration method used in the present study had sufficient sensitivity and precision. This method is simpler and less expensive than HPLC separation and requires only a centrifuge. When selecting an ultrafiltration device for the separation of proteins from carminic acid, a cylindrical device containing a modified polyethersulfone membrane gives good results, and the protein contaminants can also be detected by silver staining SDS-PAGE. This method could potentially be applied to other natural pigments and to the removal of unreacted dyes used for protein labeling.

## 4. Conclusion

A rapid and simple method was developed for the analysis of protein contaminants from carmine. Method's development focused on preventing protein aggregation under acidic protein denaturation conditions and the use of an ultrafiltration device. To prevent protein aggregation, SDS-PAGE running buffer was used for dispersion of the carmine. A cylindrical ultrafiltration device containing a modified polyethersulfone membrane provided the best separation of protein contaminants, which could then be observed as bands by SDS-PAGE. This method showed high reproducibility.

## References

[1] Dapson R. W. (2007). The history, chemistry and modes of action of carmine and related dyes. *Biotechnic and Histochemistry*.

[2] Food and Drug Administration (2014). *H. 73,100 Cochineal Extract, Carmine*.

[3] The European Parliament and the Councile of the EU (1994). The European Parliament and the Councile Directive 94/36/EC of 30 June 1994 on coluours for use in foodstuffs. *Official Journal of the European Communities*.

[4] Japanese Consumer Affairs Agency (2012). *Attention Awakening about the Cochineal Pigment*.

[5] Añíbarro B., Seoane J., Vila C., Múgica V., Lombardero M. (2003). Occupational asthma induced by inhaled carmine among butchers. *International Journal of Occupational Medicine and Environmental Health*.

[6] Lizaso M. T., Moneo I., García B. E., Acero S., Quirce S., Tabar A. I. (2000). Identification of allergens involved in occupational asthma due to carmine dye. *Annals of Allergy, Asthma and Immunology*.

[7] Ohgiya Y., Arakawa F., Akiyama H. (2009). Molecular cloning, expression, and characterization of a major 38-kd cochineal allergen. *Journal of Allergy and Clinical Immunology*.

[8] Ichi T., Koda T., Yukawa C., Sakata M., Sato H. (2002). Purified cochineal pigment and process for producing the same. *WO/2002/022743*.

[9] Shapiro A. L., Viñuela E., V. Maizel J. (1967). Molecular weight estimation of polypeptide chains by electrophoresis in SDS-polyacrylamide gels. *Biochemical and Biophysical Research Communications*.

[10] Switzer R. C., Merril C. R., Shifrin S. (1979). A highly sensitive silver stain for detecting proteins and peptides in polyacrylamide gels. *Analytical Biochemistry*.

